# Ergonomic support and workstation satisfaction among teleworkers in small and medium enterprises

**DOI:** 10.1177/10519815251386811

**Published:** 2025-10-30

**Authors:** Jérôme Prairie, Tyler Pacheco, Carol-Anne Gauthier, Maude Villeneuve, Simon Coulombe

**Affiliations:** 1 Laval University, Faculty of Social Sciences, Department of Industrial Relations, Quebec, Qc, Canada; 2 Laval University, Relief Research Chair in Mental Health, Self-management and Work, Quebec, Qc, Canada; 3 VITAM, Sustainable Health Research Center, Quebec, Qc, Canada; 4 Department of Social Sciences, Champlain College St-Lawrence, Quebec, Qc, Canada; 5 CERVO, Brain Research Center, Quebec, Qc, Canada

**Keywords:** teleworking, ergonomics, small business, medium sized business, well-being, computer, satisfaction

## Abstract

**Background:** Ergonomics can be adapted to the work-from-home context, e.g., training on adjustment, adjusted furniture provision, and professional evaluation of the ergonomic adjustment of one's workspace. However, very little research has examined the extent to which such supports have been offered to the diverse small and medium enterprise (SME) teleworkers population post-pandemic. **Objectives:** 1) Examine if, and how, i) ergonomics support provided to SME teleworkers, and ii) their satisfaction with the ergonomic adjustment of their workstation vary according to individual and organizational characteristics; 2) investigate the associations between provided ergonomics support and workstation satisfaction. **Methods:** 1162 teleworkers (616 supervisors) employed by SMEs in Canada completed an online questionnaire that included items regarding three ergonomic supports (i.e., training, equipment, evaluation) and workstation satisfaction. **Results:** Logistic and linear regression analyses indicated that several individual and organizational characteristics significantly (ps ≤ .05) predicted whether teleworkers received each form of ergonomic support (training: gender, enterprise size, union presence; equipment: supervisory role; evaluation: daily time at the computer, supervisory role) or their level of satisfaction with their workstation (union presence). Hours per week employers expected employees to telework and age significantly predicted being provided with ergonomic support (of any form) and workstation satisfaction. Linear regressions indicated that each ergonomic support was significantly related to increased workstation satisfaction. **Conclusions:** Some groups of teleworkers (e.g., women, younger workers, those in medium-sized enterprises, workers represented by a union) in SMEs seem more likely to receive ergonomic support than others. Receiving more ergonomic support cascades to more satisfaction with one's workstation.

## Introduction

In recent years, several organizations have offered workers the option of teleworking. The space in which one completes their work, traditionally arranged and provided by employers, has now evolved into multiple environments over which companies exert less influence (e.g., home offices, cafés, coworking spaces).^1–3^ This organizational change has had repercussions on the day-to-day work environment, and on possible preventive support, particularly in terms of office ergonomics.^4,5^ Alencar et al.'s systematic review revealed that teleworking conditions differ from office-based working conditions, but both can put workers at risk for musculoskeletal disorders (MSDs).^4^ MacLean et al. show that 69% of sampled employees reported working from a new home office since the onset of COVID-19. Two studies also noted a 61% to 75% increase in musculoskeletal discomfort associated with home workstations compared to office workstations.^6,7^ The risk of developing MSDs from computer work is well documented and several guidelines are promoted, whether the work is performed on the organization's premises or remotely.^6,8–13^

In sum, these findings suggest that the COVID-19 pandemic fostered the proliferation of telecommuting and an increase in MSD risks.^6,10,12^ Buomprisco et al.'s literature review identified the two most widely recognized occupational health and safety (OHS) issues for teleworkers: psychosocial risks and posture-related ergonomics risks.^3^ Concerning ergonomic risks, Wütschert et al.'s systematic review suggests that ergonomic support programs should be implemented to help workers reduce the health risks associated with telecommuting. Studies in office ergonomics, in general (i.e., most studies are not specific to home offices), show that the risk of developing MSDs can be reduced thanks to various supports (e.g., ergonomic principles training, having adapted equipment, ergonomic interventions involving specialists).^9,14–16^ In addition to preventing MSDs, ergonomic interventions have been shown to have a positive impact on organizational performance^14–16^ and quality of life at work.^14,15,17^

The very few post-COVID-19 studies on the impacts of ergonomic interventions on home workstations focus mainly on large organizations,^6,12^ which have more than 500 employees. However, small and medium-sized enterprises (SMEs) play a crucial role in the economies of several countries, such as Canada.^18–20^ In Canada, where the present study was conducted, SMEs are defined as organizations employing 1 to 99 employees (small) or 100 to 499 employees (medium).^19^ According to recent statistics, there were more than 1.21 million employing SMEs in operation in Canada at the end of 2022, representing the vast majority (99.7%) of employing businesses in the country. These enterprises employed 46.8% of the labour force of the private sector. SMEs are thought to be heavily affected by ergonomic risk factors (e.g., awkward posture, repetitive tasks, manual material handling).^18,19,21^ Due to limited financial, time, and human resources, compared to large businesses, the OHS prevention culture tends to remain weak in SMEs.^22,23^ However, a recent survey among managers from SMEs in the manufacturing sector in Québec, Canada, suggests serious prevention efforts are being made by some SMEs to address the situation. Nevertheless, the same study showed that MSDs were highly prevalent among the surveyed companies.^23^ Another study focused on MSD prevention, conducted in Ontario, Canada, among 146 SME owners and managers, highlighted their general lack of knowledge about MSD control. These owners and managers also tended to favour a traditional, reactive control approach, rather than proactive prevention strategies. These participants rarely reported equipment-related interventions (e.g., ergonomic furniture) as interventions offered in their businesses.

While such studies are relevant, they were conducted before the COVID-19 pandemic. Thus, they yield minimal information on which MSD-related prevention forms, and specifically ergonomic supports, are offered in the now widespread work-from-home context. To our knowledge, there is virtually no published research about ergonomic support offered to SME teleworkers in the post-pandemic context. However, Alencar et al. identified difficulties faced by teleworkers when attempting to set up their home office according to ergonomic criteria, such as inadequate equipment and the lack of a designated workspace.^4^ In light of this, ergonomic supports could be adapted to the work-from-home context, e.g., ergonomic adjustment training, adjusted furniture provision, and professional evaluation of the ergonomic adjustment of one's workspace.^7^ However, there is very little research concerning the extent to which such supports have been offered to the very diverse population of SME workers in the post-pandemic period, as well as to what extent these supports, when offered, have contributed (or not) to satisfactory outcomes. As an indicator of satisfactory outcomes, in the current study, we focus on employees’ levels of self-reported satisfaction with the ergonomic adjustment of their home workstation because, according to Miles and Perrewé, it is associated with lower odds of musculoskeletal pain among workers. Along the same line, satisfaction with the ergonomic training received has been associated with reduced job-induced tension and somatic complaints.^24^ In the absence of objective outcome measures, satisfaction with ergonomics thus seemed to be a relevant indicator to consider among SME employees when examining the ergonomic quality of their home workstations.

### The present study

Given the lack, to our knowledge, of post-pandemic research on ergonomic supports in the context of SME employees working from home, the current study had two exploratory objectives:

1. Examine if, and how, three forms of ergonomic support (training, equipment, evaluation) provided to SME workers, as well as their level of satisfaction with the ergonomic adjustment of their workstation, vary according to an array of individual and organizational factors.
2. Explore if forms of ergonomic support provided to SME workers are associated with their satisfaction with the ergonomic adjustment.

## Methods

### Participants

Laval University's Research Ethics Committee approved this study (Approval #2022-465/06-03-2023). Participants in the study were recruited from a large Canadian online panel (LEO). Surveys were completed on Qualtrics, an online anonymous survey platform. Participants first completed a consent form and screening questions to ensure eligibility. Participants obtained set compensation (i.e., points) determined by LEO. Eligible workers were: 1) living in Canada; 2) at least 18 years old; 3) employed by a private organization with a maximum of 499 employees; and 4) working at least 14 h/week. In March 2023, 2500 participants were sampled. Many participants were excluded because they were not teleworking (*n* = 1138) or failed the survey's embedded attention check (*n *= 230). Our final analytical sample had 1162 participants (including 616 supervisors).

### Measures

Participants answered individual- (e.g., gender, age, hours teleworked) and organizational-level (e.g., enterprise size,^19^ union presence) measures. They also answered four questions specific to home office ergonomics: 1) workstation satisfaction (‘My workstation [chair, work surface, monitor, keyboard, mouse and ambient light] is well adjusted to my personal characteristics’; answered from 1 [*Strongly Disagree*] to 5 [*Strongly Agree*]), 2) access to ergonomic training (‘I have already taken a training course [e.g., webinar, conference, workshop] in office ergonomics to help me adjust my teleworkstation’), 3) received ergonomic equipment provisions (‘My company has provided or paid for all the equipment [adjustable chair, adjustable desk, adjustable monitor, mouse and keyboard] needed to set up my teleworkstation’), and 4) provided with ergonomic evaluation of workstation (‘A person paid by my employer validated my teleworkplace adjustment [e.g., on-site or virtual ergonomist, OHS advisor, other]’). The latter three items reflect forms of ergonomic supports and are answered 0 (*No*) or 1 (*Yes*). For brevity, these supports are respectively referred to as ‘training,’ ‘equipment,’ and ‘evaluation’ hereafter.

### Data analysis

Analyses were conducted in SPSS version 28.0. Low proportions of workers (<5%) in certain groups (non-binary workers, living in the Territories) may have affected the conducted analyses. Due to this, such groups were omitted from the main analyses. Time employed at one's company was removed as a predictor from the regression models due to multicollinearity concerns (Tolerance = .08; VIF = 12.81 and 12.89).

For Objective 1, logistic regression models were conducted for ergonomic supports (training, equipment, evaluation), and a linear regression model was performed for workstation satisfaction. The regression models contained two blocks: the first containing the individual-level variables and the second containing the organizational-level variables. For brevity, models containing both individual- *and* organizational-level variables are reported. Nagelkerke and adjusted *R^2^* were reported on, respectively, for the logistic and linear regression models. The Hosmer and Lemeshow statistic was also considered for determining the fit of the logistic regression models. As the results of these statistics all showed non-significant results (indicating good fit), thus coinciding with the omnibus *F*-statistic results of the regression analyses, we solely report the *F*-statistic for brevity.

For Objective 2, we follow the same protocol used for conducting the linear regression models for Objective 1. This is with the exception that a third block is added to the models with one, or all, the ergonomic supports. The third model is reported on, which controls for all the individual- and organizational-level variables.

## Results

### Descriptive statistics

Regarding the workers themselves, as shown in Table 1, most participants were between 35 and 44 years of age (44.45 ± 12.21). Most were men, English speakers, born in Canada, White, and residing in Ontario. Most participants also reported that they were working full-time, expected to telework more than 24 h per week (20.40 ± 15.49), spending at least one consecutive or four non-consecutive hours at a computer for work, employed at least five years at their company, supervising at least one employee, and within a Skill Level A job (which is characteristic of a management-level job or a job requiring a university education). Regarding the employing enterprises, as shown in Table 2, most enterprises were small (10-99 employees), operated in the professional, scientific, and technical services industry, and did not have a union.

**Table 1.** Worker characteristics.

Variable	N (%)
**Age (in years)**	
18 to 24	51 (4.40)
25 to 34	242 (20.90)
35 to 44	295 (25.40)
45 to 54	276 (23.70)
55 to 64	257 (22.20)
≥ 65	41 (3.50)
**Gender**	
Man	661 (56.90)
Woman	492 (42.30)
Non-binary	8 (0.70)
Missing response/Prefer not to say (%)	0.10
**First Language**	
French	212 (18.30)
English	817 (70.30)
Other	133 (11.40)
**Born in Canada**	
No	284 (24.40)
Yes	878 (75.60)
**Ethnicity**	
White	778 (67.00)
One ethnic minority identity	305 (26.30)
Mixed Race	79 (6.80)
**Residing Province or Territory**	
British Columbia	173 (14.90)
Ontario	489 (42.10)
Prairies^1^	191 (16.40)
Atlantic Provinces^2^	69 (5.90)
Québec	240 (20.70)
Territories^3^	0 (0.00)
**Working Full-Time** ^4^	
No	164 (14.10)
Yes	998 (85.90)
**Hours/week employers expect to be teleworked**	
> 0 to ≤ 8 h	400 (34.50)
> 8 to ≤ 24 h	310 (26.70)
> 24 h	452 (38.90)
**Daily work time at the computer**	
< 30 min. consecutive or < 60 min. non-consecutive	90 (7.80)
≥ 30 min. to < 60 min. consecutive or ≥ 1 to < 4 h non-consecutive	197 (17.00)
≥ 1 consecutive or ≥ 4 h non-consecutive	874(75.30)
**Time employed at company**	
< 1 year	23 (2.00)
≥ 1 to < 5 years	424 (36.50)
≥ 5 years	715 (61.50)
**Have 1** **+** **employee(s) under direct supervision**	
No	546 (47.00)
Yes	616 (53.00)
**Job Type (NOC Occupational Skill Level)**	
Skill Level A (Management; req. university education)	740 (63.70)
Skill Level B (Jobs req. college education)	242 (20.80)
Skill Level C (Jobs req. high school education)	77 (6.60)
Skill Level D (Jobs req. on-the-job training)	49 (4.30)
Other	54 (4.70)

*Note*: ^1^ Alberta, Manitoba, Saskatchewan; ^2^ New Brunswick, Newfoundland and Labrador, Nova Scotia, Prince Edward Island; ^3^ Nunavut, Yukon, Northwest Territories; ^4^ A worker is considered full-time if they work greater than, or equal to, 30 hours per week compared to a part-time worker working less than 30 hours per week.

**Table 2.** Enterprise characteristics.

Variable	N (%)
**SME Size**	
Very small (0 to 9 people)	295 (25.40)
Small (10 to 99 people)	584 (50.30)
Medium (100 to 499 people)	282 (24.30)
**Industry Sector**	
Retail trade	67 (5.70)
Other services, except public administration	23 (1.90)
Professional, scientific and technical services	297 (25.60)
Arts, entertainment, and recreation	50 (4.30)
Finance and insurance	60 (5.20)
Health care and social assistance	83 (7.20)
Construction	61 (5.30)
Accommodation and food services	18 (1.50)
Educational services	53 (4.50)
Wholesale trade	30 (2.60)
Manufacturing	75 (6.40)
Information and cultural industries	6 (0.50)
Transportation and warehousing	28 (2.40)
Agriculture, forestry, fishing and hunting	9 (0.70)
Management of companies and enterprises	14 (1.20)
Real estate and rental and leasing services	25 (2.20)
Administrative and support, waste management and remediation services	21 (1.80)
Public administration	12 (1.00)
Mining, oil and gas extraction	12 (1.00)
Utilities	8 (0.70)
Other	117 (10.00)
Multiple industries	96 (8.20)
**Presence of a Union**	
No	1029 (88.60)
Yes	133 (11.40)

Note: ^1^ = industry sectors containing less than 5% of the total sample were merged into the “Other” category for the main analyses.

Participants scored moderately high (3.82 ± 1.00; scale range: 1 [*Strongly Disagree*] to 5 [*Strongly Agree*]) in their satisfaction with their telecommuting workstation and its suitability to their personal characteristics. Of the 1162 participants, 38.30% had received all the equipment they needed to telework, 30.00% had received training to adjust their home workstation, and 15.90% had an employer who paid an ergonomics or health and safety specialist to validate their ergonomic adjustments at home.

### Main analyses

Below, we discuss the associations between individual- and organizational-level variables, ergonomic supports, and workstation satisfaction. Unless we specify that the relationship is a “trend” (*p* ≤ .10), the presented associations were significant at *p* ≤ .05.

### Predicting ergonomic support presence

Logistic regressions (Table 3) were conducted to investigate the associations between individual- and organization-level variables and the presence of diverse forms of ergonomic supports (i.e., training, equipment, evaluation) (Models 1-3) and on the presence of any of these supports (Model 4). The ergonomic support models were all significant (training: χ^2^[33] = 89.26, *p* < .001; equipment: χ^2^[33] = 93.14, *p* < .001; evaluation: χ^2^[33] = 181.22, *p* < .001; binary presence of any form of support: χ^2^[33] = 78.86, *p* < .001). Nagelkerke's R indicates that 11.60% (training), 11.50% (equipment), 26.60% (evaluation), and 9.70% (binary presence of any form of support) of the variance were explained. The models correctly classified 61.80% to 86.60% of the cases.

**Table 3.** Logistic regression analyses.

Variable	Model 1: Training ergonomic support		Model 2: Equipment ergonomic support		Model 3: Evaluation ergonomic support		Model 4: Ergonomic support (Binary)	
	*B*	* Odds ^a^* *[95% CI]*	*B*	* Odds ^a^* *[95% CI]*	*B*	* Odds ^a^* *[95% CI]*	*B*	* Odds ^a^* *[95% CI]*
**Constant**	−1.06*	0.35	−0.48	0.62	−0.89	0.41	0.36	1.43
***Individual-level variables***								
**Hours/week employers expect to be teleworked**								
≥ 0 to ≤ 8 h (Reference)	–	–	–	–	–	–	–	–
> 8 to ≤ 24 h	0.38*	1.47[1.02,2.10]	0.38*	1.46[1.03,2.08]	0.54*	1.71[1.10,2.67]	0.42*	1.52[1.08,2.13]
> 24 h	0.01	1.01[0.70,1.45]	0.34*	1.40[1.00,1.97]	−0.35	0.70[0.43,1.17]	0.27^t^	1.32[0.95,1.82]
**Daily work time at the computer**								
< 30 min. consecutive or < 60 min. non-consecutive (Reference)	–	–	–	–	–	–	–	–
≥ 30 min. to < 60 min. consecutive or ≥ 1 to < 4 h non-consecutive	0.41	1.51[0.80,2.86]	0.35	1.41[0.79,2.51]	0.42	1.52[0.77,2.99]	0.26	1.29[0.73,2.28]
≥ 1 consecutive or ≥ 4 h non-consecutive	0.23	1.25[0.70,2.25]	−0.22	0.81[0.48,1.37]	−0.82**	.44[0.23,0.85]	−0.13	.88[0.53,1.46]
**Age (in years)**								
18 to 24 (Reference)	–	–	–	–	–	–	–	–
25 to 34	−1.02**	.36[0.17,0.79]	−0.69^t^	0.50[0.24,1.06]	−1.27**	0.28[0.12,0.68]	−0.99*	0.37[0.17,0.84]
35 to 44	−0.95*	.39[0.18,0.83]	−0.51	0.60[0.29,1.24]	−1.73***	0.18[0.07,0.43]	−0.73^t^	0.48[0.22,1.07]
45 to 54	−0.84*	.43[0.20,0.94]	−0.48	0.62[0.29,1.30]	−1.35**	0.26[0.11,0.63]	−0.82*	0.44[0.20,0.99]
55–64	−0.98*	.38[0.17,0.82]	−0.92*	.40[0.19,0.85]	–1.67***	0.19[0.08,0.47]	–1.11**	0.33[0.15,0.74]
≥ 65	–1.04*	.35[0.12,1.00]	–1.02*	.36[0.14,0.97]	–1.52*	0.22[0.06,0.79]	–1.03*	0.36[0.13,0.97]
**Women (vs. Men)**	0.36*	1.43[1.06,1.93]	0.13	1.14[0.86,1.52]	0.10	1.10[0.74,1.65]	0.19	1.21[0.91,1.59]
**First language**								
English (Reference)	–	–	–	–	–	–	–	–
French	0.29	1.33[0.74,2.41]	−0.64*	0.53[0.30,0.92]	0.22	1.25[0.50,3.09]	−0.06	0.94[0.56,1.60]
Other	−0.36	0.70[0.41,1.20]	−0.35	0.70[0.43,1.16]	−0.42	0.66[0.33,1.30]	−0.33	0.72[0.44,1.18]
**Immigrant (vs. Born in Canada)**	0.22	1.24[0.81,1.89]	0.70***	2.02[1.34,3.03]	0.78**	2.18[1.29,3.69]	0.63**	1.89[1.25,2.84]
**Ethnic identity**								
White (Reference)	–	–	–	–	–	–	–	–
One ethnic minority identity	0.30	1.34[0.90,2.01]	−0.14	0.87[0.60,1.28]	0.03	1.03[0.62,1.72]	−0.01	1.00[0.68,1.45]
Mixed race	0.04	1.04[0.58,1.89]	−0.36	0.70[0.39,1.25]	0.23	1.26[0.60,2.65]	−0.44	.65[0.37,1.12]
**Residing province or territory**								
Ontario (Reference)	–	–	–	–	–	–	–	–
British Columbia	−0.35	0.71[0.45,1.12]	0.17	1.18[0.79,1.77]	−0.05	0.95[0.55,1.64]	0.05	1.05[0.71,1.55]
Prairies	0.54**	1.71[1.15,2.53]	0.35^t^	1.42[0.97,2.09]	0.56*	1.76[1.07,2.89]	0.48*	1.61[1.10,2.37]
Atlantic Provinces	0.06	1.07[0.57,2.01]	0.60*	1.83[1.00,3.35]	0.62	1.87[0.86,4.05]	0.32	1.38[0.76,2.48]
Québec	−0.24	0.79[0.44,1.40]	0.69**	1.98[1.16,3.39]	−0.75	0.47[0.19,1.17]	0.27	1.30[0.78,2.18]
**Working full-time (vs. Not)**	−0.02	0.98[0.62,1.54]	−0.05	0.95[0.62,1.46]	−0.20	0.82[0.47,1.42]	−0.24	0.79[0.52,1.19]
**Have employee(s) under direct supervision (vs. Do not have)**	0.06	1.06[0.78,1.45]	0.39**	1.48[1.10,1.98]	0.79***	2.21[1.43,3.41]	0.18	1.20[0.90,1.59]
**Job Type (NOC occupational skill level)**								
Skill Level A (Management; req. university education) (Reference)	–	–	–	–	–	–	–	–
Skill Level B (Jobs req. college education)	−0.20	0.82[0.55,1.21]	−0.04	0.96[0.67,1.38]	0.09	1.10[0.65,1.87]	−0.12	0.89[0.62,1.26]
Skill Level C (Jobs req. high school education)	0.05	1.05[0.58,1.91]	0.30	1.36[0.76,2.41]	0.41	1.51[0.72,3.17]	0.04	1.04[0.59,1.83]
Skill Level D (Jobs req. on-the-job training)	0.75^t^	2.13[0.98,4.60]	0.83*	2.28[1.05,4.97]	0.54	1.72[0.66,4.51]	0.67^t^	1.95[0.89,4.24]
Other	0.35	1.42[0.66,3.03]	−0.33	0.72[0.32,1.62]	−0.51	0.60[0.17,2.19]	0.22	1.24[0.61,2.55]
***Organization-level Variables***								
**SME size**								
Very small (0 to 9 people) (Reference)	–	–	–	–	–	–	–	–
Small (10 to 99 people)	0.32^t^	1.38[0.94,2.02]	−0.10	0.90[0.64,1.27]	0.18	1.20[0.71,2.03]	0.13	1.14[0.82,1.58]
Medium (100 to 499 people)	0.69***	2.00[1.31,3.05]	0.16	1.17[0.80,1.73]	0.54^t^	1.72[0.98,2.99]	0.59**	1.80[1.23,2.64]
**Industry sector**								
Professional services (Reference)	–	–	–	–	–	–	–	–
Retail	0.09	1.10[0.61,1.99]	−0.71*	0.49[0.27,0.91]	0.24	1.27[0.61,2.65]	−0.22	0.80[0.46,1.40]
Healthcare and social assistance	0.27	1.31[0.78,2.19]	−0.21	0.81[0.48,1.35]	0.22	1.24[0.63,2.45]	−0.10	0.91[0.56,1.48]
Construction	0.01	1.01[0.55,1.86]	0.16	1.17[0.66,2.07]	−0.24	0.79[0.35,1.77]	0.07	1.08[0.61,1.89]
Manufacturing	−0.10	0.90[0.51,1.61]	0.21	1.23[0.74,2.07]	0.31	1.37[0.67,2.82]	0.06	1.07[0.64,1.77]
Other/Multiple industry(ies)	0.09	1.09[0.67,1.79]	0.62**	1.86[1.17,2.94]	0.93***	2.54[1.44,4.49]	0.44^t^	1.55[0.97,2.48]
**Presence of a union (vs. No presence)**	0.65**	1.91[1.25,2.93]	0.34	1.40[0.92,2.14]	0.29	1.34[0.77,2.31]	0.33	1.39[0.90,2.15]

*** *p* ≤ .001; ** *p* ≤ .01; * *p* ≤ .05; ^t^ *p* ≤ .10.

*Note*: ^a^ = odds ratios are only provided when the predictor is at least trending in the model.

Regarding Model 1, workers expected to telework more than eight to 24 h/week (vs. ≤ eight hours) by their employer were more likely to have taken a training course in office ergonomics. Workers 25 years old or more were less likely to have completed ergonomics training. Women (vs. men) and workers in the Prairies (vs. Ontario) were more likely to have received such training. Medium-sized (vs. very small) SMEs and the presence of a union (vs. no presence) were associated with higher odds of undergoing such training.

Regarding Model 2, workers who were expected to telework more than eight to 24 h/week or more than 24 h (vs. ≤ eight hours) were more likely to have employer-provided or paid-for equipment. Although workers between the ages of 25-34, 55-64, or 65+ (vs. 18-24) were less likely to have been provided with ergonomic equipment, the result regarding 25–34-year-olds was only trending. Workers whose first language was French (vs. English) were less likely to have reimbursed equipment; immigrants (vs. those born in Canada) were more likely. While workers in the Prairies, Atlantic provinces, or Québec (vs. Ontario) were more likely to have such equipment, the result for the Prairies is trending. Those who supervised other employees (vs. not) or worked in a job that required on-the-job training (i.e., occupational skill level-D; vs. skill level-A) were more likely to have obtained equipment provided by their employer. Retail (vs. professional services) workers were less likely to have such equipment; those in other or multiple industries were more likely.

Regarding Model 3, workers expected to telework more than eight to 24 h/week (vs. ≤ eight hours) by their employer were more likely to have received a workstation evaluation paid for by their employer. Those who spent one or more consecutive hour(s) or four or more non-consecutive hours (vs. < 30 min. consecutive or <60 min. non-consecutive hours) per day at their computer were less likely to have had such an evaluation. Similarly, those older than 18 to 24 were less likely to have received an evaluation. Immigrants (vs. those born in Canada), workers living in the Prairies (vs. Ontario), and supervisors (vs. those not supervising anyone) were more likely to have received such an evaluation. Those in medium-sized SMEs (vs. very small) or working in other/multiple industries (vs. professional services) were more likely to have received a workstation evaluation; however, the result for medium-sized SMEs was trending.

Regarding Model 4, workers expected to telework more than eight to 24 h/week or more than 24 h (vs. ≤ eight hours) were more likely to be given at least one of the three ergonomic supports (training, equipment, evaluation). However, the results for workers expected to telework more than 24 h are trending. Those older than 25 were less likely to have received ergonomic support; however, the results for 35 to 34-year-olds were trending. Immigrants (vs. those born in Canada), workers living in the Prairies (vs. Ontario), and those who worked in a job that required on-the-job training (i.e., occupational skill level-D; vs. skill level-A) were more likely to have received ergonomic support. However, the result for workers in a skill level-D job type was trending. Those in medium-sized SMEs (vs. very small) or other/multiple industries (vs. professional services) were more likely to have received ergonomic support; however, the result for other/multiple industries was trending.

### Predicting workstation satisfaction

Simple linear regression (Table 4) was conducted to investigate the extent to which individual- and organization-level variables predict workstation satisfaction. The model was significant (*F*[33, 1010] = 2.12, *p* < .001), with the predictors explaining 3.40% of the variance in workstation satisfaction. Those expected to work more than eight to 24 h/week or more than 24 h/week (vs. ≤ eight hours) were found to have an increased satisfaction score by 0.28 and 0.34 points, respectively. Although 25- to 34-year-olds (−0.49 points) and 35- to 44-year-olds (−0.32 points) (vs. 18-24) were predicted to have decreased satisfaction scores, the results regarding 35- to 44-year-olds were only trending. Lastly, the presence of a union was significantly associated with a 0.20-point increase in workstation satisfaction.

**Table 4.** Linear regression analysis.

Variable	Dependent variable: Workstation satisfaction
	*B Coeff.*
**Constant**	3.78***
***Individual-level Variables***	
**Hours/week employers expect to be teleworked**	
≥ 0 to ≤ 8 h (Reference)	–
> 8 to ≤ 24 h	0.28***
> 24 h	0.34***
**Daily work time at the computer**	
< 30 min. consecutive or < 60 min. non-consecutive (Reference)	-
≥ 30 min. to < 60 min. consecutive or ≥ 1 to < 4 h non-consecutive	0.11
≥ 1 consecutive or ≥ 4 h non-consecutive	0.13
**Age (in years)**	
18 to 24 (Reference)	–
25 to 34	−0.49**
35 to 44	−0.32 ^t^
45 to 54	−0.28
55–64	−0.16
≥ 65	−0.22
**Women (vs. Men)**	0.05
**First Language**	
English (Reference)	–
French	−0.11
Other	−0.01
**Immigrant (vs. Born in Canada)**	−0.03
**Ethnic Identity**	
White (Reference)	–
One ethnic minority identity	−0.12
Mixed race	−0.11
**Residing Province or Territory**	
Ontario (Reference)	–
British Columbia	0.02
Prairies	0.11
Atlantic Provinces	0.16
Québec	0.08
**Working Full-Time (vs. Not)**	0.02
**Have employee(s) under direct supervision (vs. Do not have)**	−0.10
**Job Type (NOC Occupational Skill Level)**	
Skill Level A (Management; req. university education) (Reference)	–
Skill Level B (Jobs req. college education)	0.02
Skill Level C (Jobs req. high school education)	−0.15
Skill Level D (Jobs req. on-the-job training)	−0.09
Other	0.02
***Organization-level Variables***	
**SME Size**	
Very small (0 to 9 people) (Reference)	–
Small (10 to 99 people)	0.09
Medium (100 to 499 people)	0.09
**Industry Sector**	
Professional services (Reference)	–
Retail	−0.14
Healthcare and social assistance	−0.01
Construction	0.04
Manufacturing	−0.01
Other/Multiple industry(ies)	−0.04
**Presence of a Union (vs. No presence)**	0.20*

*** *p* ≤ .001; ** *p* ≤ .01; * *p* ≤ .05; ^t^ *p* ≤ .10.

### Predicting workstation satisfaction from ergonomic supports

Four simple linear regressions (Table 5) were conducted to explore how the individual ergonomic supports, a) considered individually (Models I-III) or b) together in the same model (Model IV), predicted workstation satisfaction. All four models were significant (Model I: *F*[34, 1009] = 2.80, *p* < .001; Model II: *F*[34, 1009] = 3.04, *p* < .001; Model III: *F*[34, 1009] = 2.46, *p* < .001; Model IV: *F*[36, 1007] = 3.47, *p* < .001), and explained between 4.50% and 7.80% of the variance in workstation satisfaction. When considered individually, all three individual ergonomic supports were significantly related to increased workstation satisfaction, with training being the most associated and evaluation the least. When the three forms of support were considered together in the same model, training, and equipment were still significantly related to workstation satisfaction, but evaluation was not.

**Table 5.** Predicting workstation satisfaction from ergonomic support variables.

Ergonomic Supports	Model I	Model II	Model III	Model IV
	*B Coeff.*	*B Coeff.*	*B Coeff.*	*B Coeff.*
Training	0.38***	–	–	0.31***
Equipment	–	0.35***	–	0.29***
Evaluation	–	–	0.32***	−0.01

*** *p* ≤ .001.

*Note*. All variables from Table 1 were included as controls.

## Discussion

This exploratory study provides, to our knowledge, the first available empirical insights into the ergonomic support SME teleworkers receive for their at-home workstations. This is a significant contribution to the scientific literature, as most existing studies on this topic conducted since the pandemic have focused on large organizations, where the organizational context differs significantly (e.g., more financial resources and ergonomic expertise available). Regarding Objective #1, the three ergonomic supports (training, equipment, evaluation) and workstation satisfaction were associated with many individual and organizational covariates. Although some of these covariates were different based on the ergonomic support considered, irrespective of individual categories within the explored predictors, we found that several individual (gender, age, immigration status, hours/week teleworked) and organizational (company size, supervisorial role, union presence) characteristics predicted the presence of one or more organizational support(s). We also found individual (hours/week teleworked, age) and organizational (union presence) characteristics that predicted workstation satisfaction. Regarding Objective #2, the presence of each form of ergonomic support was positively associated with workstation satisfaction. However, when considered together, only training and equipment were significantly associated with ergonomic support.

Studies conducted during the COVID-19 pandemic have shown that working from home can be hazardous to musculoskeletal health, particularly due to the presence of makeshift workspaces and the use of non-ergonomic equipment.^4^ For example, Snodgrass et al. found that laptops replaced desktop computers for teleworkers, leading to poor posture due to non-ergonomic setups.^25^ It is clear, from two recent reviews, that the prevention of MSDs requires employers to take specific actions with teleworkers regarding managing workload and offering physical and psychological support.^4,10^ Our study reveals that a relatively small proportion of teleworkers in SMEs (38%) received support in the form of ergonomic equipment provision. This lack of adaptable workstation equipment, such as adjustable monitors, an external keyboard/mouse, an adjustable desk, and an adjustable chair, can lead to the adoption of strained postures that affect the neck, wrists, shoulders, and back.^4,8,9^ In addition, studies show that it is necessary to provide teleworkers with alternative means to ensure effective OHS prevention during the transition from the office to the home office. The lack of equipment, proper training, and access to a trained ergonomist to address any unique issues faced in setting up the workstation has increased MSD-related discomforts.^6,7,13,26^ The results of our study indicate that access to training or specialist assistance is even lower than the equipment offered to SME teleworkers (30% and 16%, respectively). Our results suggest that SMEs in Canada seeking to transition to teleworking while ensuring the musculoskeletal health and well-being of their workers should implement more comprehensive ergonomic support.

### Who receives ergonomic support and why?

As previously mentioned, women received more ergonomic support training. Potential explanations include the fact that women tend to adopt positions that are less ergonomic than men, especially when using non-adjustable workstations. They are also at higher risk of developing MSD when sharing workstations with men since these are more likely to be adjusted for the man. Such scenarios can lead to the exacerbation of postural constraints for women.^6^ These negative impacts on working women's health could explain why our results show that women obtained more ergonomic training; it potentially reflects the fact that they are more likely than men to have sought such training. In the case of 18- to 24-year-old workers, it is plausible that these younger workers are just entering the organization, and that ergonomic supports were integrated into their onboarding/training process. More research is needed, however, to explore this finding as some studies suggest that younger workers are less likely to be involved in health and safety prevention programs than older coworkers in various fields (e.g., agriculture, food service).^27^ Similarly, future research is needed to help unpack the association found between some ergonomic supports and immigrant status, as the current knowledge based on ergonomic-related themes among immigrants is too scarce to identify plausible mechanisms underlying this association.

Regarding organizational characteristics, company size, supervisory role, and union presence all predict the presence of some ergonomic assistance. The smaller the company, the less likely it was to provide ergonomic support. Occupational health and safety preventive measures are more challenging to implement in SMEs, notably due to issues related to the allocation and preventive skills of human and technical resources,^10,20^ financial obstacles to invest in prevention,^19,20^ and union absence.^20^ In addition, Wütscher et al. report that some companies do not offer workers ergonomic support, even when requested by them, either because of the absence of an ergonomic program or because they do not consider ergonomic support an essential need.^10^ Given that unionization rates are lower among SMEs and that SMEs experience more difficulties in implementing preventive measures,^20^ it is interesting to note that union presence favours ergonomic prevention.

Knowledge of companies’ support options could explain why supervisors were more likely to self-report obtaining employer-paid equipment, as well as an evaluation from an ergonomic expert. Wütscher et al. suggest that most teleworkers would not want their employer to interfere in their home under the pretext of health and safety inspections, nor would employers wish to impose themselves.^10^ However, as supervisors are hierarchically higher in the organization than most workers, they may be more comfortable with such inspections. Our study suggests inequalities between SME employees, as supervisors appear to be more likely to receive paid forms of prevention.

The primary variable associated with higher workstation satisfaction was time spent at the computer. This result is consistent with the fact that workers who spend more time at a computer are more likely to have received ergonomic support. Logically, the increase in ergonomic prevention should be associated with time spent at one's workstation, since the onset of an MSD is associated with modulators of duration, frequency, and intensity of exposure to the risk factor.^28^ Longer duration of deskwork may cause higher exposure to MSD risk factors (repetitive movements, mechanical pressure, awkward and/or maintaining static posture). The exposure may lead to discomfort and injury, particularly in workers’ backs, necks, and upper limbs. Such health problems may have prompted teleworkers and their employers to seek solutions, or their doctors may have referred them for office ergonomics support.

Understanding which characteristics significantly predict the presence of ergonomic support and workstation satisfaction has the potential to help target teleworking populations that require greater attention regarding workstation ergonomics. Providing greater ergonomic support may help prevent adverse health outcomes (e.g., MSDs) and improve workplace outcomes (e.g., performance).

### Predicting workstation satisfaction from ergonomic supports

Our results show that each type of ergonomic support offered to SME teleworkers (i.e., training, equipment, professional evaluation) was associated with self-reported workstation satisfaction. This result is reminiscent of other studies investigating the positive impacts of ergonomic interventions on outcomes (e.g., discomfort, injury) related to computer workstations.^8,10–12^ However, it is essential to mention that these studies use more objective and detailed indicators than satisfaction to measure the impact of ergonomic interventions. For example, Emerson et al. evaluated workstation quality using photographs and measurements, while Kotowski et al. included specific questions in their questionnaire regarding the type of equipment used and the discomfort experienced in each body region.^8,12^ There are also ergonomic risk checklists, such as the Rapid Office Strain Assessment, designed to quantify risks associated with computer work; however, their use requires specialized ergonomic expertise.^29^ Given that the current study was part of a larger study, such a detailed analysis of more specific ergonomic risk was not feasible (i.e., to maintain the survey at an acceptable length).

When all three explored ergonomic supports are considered simultaneously, training and equipment play dominant roles in predicting workstation satisfaction, whereas evaluation is no longer a significant predictor. The fact that training is a predictor is not surprising given prior research. For example, according to Robertson et al., the knowledge acquired through training enables individuals to appropriately modify their workstations, thereby reducing the risk of MSDs and enhancing organizational efficiency.^15^ However, other studies suggest a more complex and tailored approach involving professionals to achieve quality results.^8,30^ Specialists use office ergonomic standards recognized in the scientific literature,^2,5^ and these are often available and promoted in various accessible formats (e.g., training materials, articles, videos) in workplaces. A specialist's role includes providing training-related information and making modifications (e.g., proposing adapted equipment). In other words, if workers completed training with a specialist, then it is possible that actual changes were made during this training, which might explain the positive outcomes. Thus, a co-occurrence of certain support forms (or shared variance between them) may explain the pattern of findings found in this study.

### Limitations and future directions

This study has several limitations. First, the self-reported nature of the measures may have biased our results. The items used to assess the types of ergonomics support were not validated through psychometric analysis, which may compromise the quality of the measurement. Future research with more objective and detailed indicators (to complement satisfaction) is needed, as well as psychometric evaluation of the items in comparison with criterion measures (e.g., on-site ergonomic evaluation). However, given that the items had simple *yes-or-no* answers regarding whether participants had received relatively obvious and concrete forms of support or not, it is thought that these items were easily answerable by participants, with limited possibility for misinterpretation, and with adequate face validity. Second, SMEs may be less likely than larger enterprises to have standardized health and safety protocols. Our study draws conclusions about the ergonomic support provided to workers by SMEs, but it is possible that external professionals (e.g., doctors) mandated the ergonomic support. Third, this study used a select few individual and organizational characteristics to predict the presence of ergonomic support. Future works should explore several other variables, notably in the fields of ergonomics, OHS and organizational psychology (e.g., anthropometric and psychosocial profile, years of teleworking, office design, psychosocial risk factors, safety climate, individual coping strategies, etc.). The non-inclusion of these other relevant and potentially powerful explanatory factors may explain why the effect sizes of the models (percentage of explained variance) were modest in our results. Lastly, certain variables emerged as statistically significant (e.g., immigrant status, place of residence), but their interpretation remains limited due to the lack of literature on these topics. While these limitations affect the current study, given the extremely limited amount of research conducted thus far among SME teleworkers since the end of the pandemic, the findings from this exploratory study are noteworthy because they have the potential to stimulate future research in these areas.

## Conclusion

This study presents a portrait of the ergonomic support provided to SME teleworkers. Few teleworkers in these organizations receive the ergonomic support required to protect their health and well-being. The provision of ergonomic support in SMEs is influenced by workers’ individual characteristics, organizational characteristics, and the nature of the work being performed. In addition, satisfaction with one's workstation is related to the form of ergonomic support received. Finally, workers in (very) small enterprises received the least ergonomic support. Given the importance of the SME workforce and the ongoing organizational transitions to teleworking, future studies are strongly encouraged to find ways to reinforce ergonomic practices among teleworkers in SMEs. Recognizing that several obstacles exist to prevention, it is necessary to review how policies and interventions are designed and implemented.
